# Sleep Disturbances in Amyotrophic Lateral Sclerosis and Prognostic Impact—A Retrospective Study

**DOI:** 10.3390/life14101284

**Published:** 2024-10-11

**Authors:** Filipa Silva, Joelma Silva, Sofia Salgueira, Ana Mendes, Elsa Matos, Bebiana Conde

**Affiliations:** 1 Pulmonology Department, Unidade Saúde Local de Trás-os-Montes e Alto Douro, 5000-508 Vila Real, Portugal; jccsilva@chtmad.min-saude.pt (J.S.); sgsalgueira@chtmad.min-saude.pt (S.S.); amendes@chtmad.min-saude.pt (A.M.); elsafm@chtmad.min-saude.pt (E.M.); bfconde@chtmad.min-saude.pt (B.C.); 2 Instituto de Investigação e Inovação em Saúde (I3S), 4200-135 Porto, Portugal; 3 Escola Superior de Saúde, Universidade de Trás-os-Montes e Alto Douro (UTAD), 5000-801 Vila Real, Portugal

**Keywords:** amyotrophic lateral sclerosis, lung function tests, polysomnography, sleep-disordered breathing

## Abstract

Amyotrophic lateral sclerosis (ALS) is a neurodegenerative disease associated with sleep disturbance, namely insomnia and sleep-disordered breathing. This study aims to evaluate the overall sleep characteristics of ALS patients, their association with lung function tests, and possible predictive survival factors. We conducted a retrospective observation study among ALS patients monitored during a pulmonology consultation. Type one polysomnography (PSG) and lung function tests were performed once the patients presented with sleep-related symptoms, and the relationship between their parameters was assessed, as well as a survival analysis. We included 35 patients, with an overall diminished sleep efficiency, a partially conserved forced vital capacity (FVC), and low maximal inspiratory pressure (MIP). A positive correlation between FVC and REM sleep percentage was observed. A survival analysis showed that a normal rapid eye movement (REM) sleep percentage and respiratory disturbance index (RDI) ≥ 15/h were independent predictors of survival. We observed a trend for higher sleep quality in patients with conserved lung function. A better sleep quality was associated with a higher survival. Obstructive events (reduced or absence of airflow associated with continued or increased inspiratory effort) did not seem to impact survival.

## 1. Introduction

Amyotrophic lateral sclerosis (ALS) is the most common motor neuron disease in adults [1]. It is a progressive neurodegenerative disease that impacts the central and peripheral nervous system and is characterized by the degeneration of inferior and superior motor neurons. This condition has a high phenotypic heterogeneity [2]. 

The major risk factors for the development of ALS known to date are genetic predisposition, epigenetic changes, aging, and cumulative environmental exposures [3,4,5]. ALS patients suffer from muscular atrophy, weakness, fatigue, and swallowing disorders. This assembly often leads to respiratory insufficiency, progressive functional loss of independence, and death [5,6]. The incidence and prevalence of ALS is variable, according to epidemiology studies. Chio et al. reported an average incidence in Europe of 2.8/100,000 and 1.8/100,000 in North America and an average prevalence of 5.4/100,000 in Europe and 3.4/100,000 in North America [5]. Considering its distribution by gender, in Europe, this disease affects men more frequently, with an incidence rate ratio of 1.4 (male/female), according to Logroscino et al. [7]. ALS is, by definition, a progressive disease, with an average survival of 20 to 48 months. However, 10 to 20% of patients exhibit a survival greater than 10 years [8]. The disease has a generally late onset, although juvenile cases of ALS (before 25 years old) represent approximately 1% and cases before 45 years old account for approximately 10% [2]. When symptoms of ALS are linked to dysfunction in the bulbar segment (such as dysphagia, sialorrhea, and dysarthria), patients are said to have a bulbar onset; in contrast, patients with a spinal onset usually first experience symptoms in their extremities, which are linked to dysfunction in the cervical, thoracic, or lumbosacral regions (such as stumbling or grasp weakness) [9].

Many factors may cause sleep disturbances in ALS patients. Some may be a result of reduced mobility, muscle cramps, pain, and swallowing disturbances [10,11,12]. Depression and anxiety may also lead to significant insomnia [13]. Early and mid-sleep disturbances may also occur due to restless leg periodic limb movement disorder and increased myoclonic activity, seen in motor neuron disease due to its underlying neuropathy [14,15,16]. In this disease, the sleep structure may be altered, namely with a decrease in total sleep time, its efficiency, rapid eye movement (REM) sleep percentage, and deep wave sleep percentage [13,17,18,19,20,21]. 

The presence of obstructive events (defined by reduced or absence of airflow associated with continued or increased inspiratory effort) [22] during sleep, although common, do not often lead to oxygen desaturation [17]. While some research indicates that obstructive sleep apnea (OSA) occurrences during sleep are rare in ALS patients [19], other investigations reveal that OSA events are present in ALS patients [23]. 

Hypoventilation, however, seems to be the main cause of nocturnal hypoxemia [13,24]. One of the main causes of sleep-induced hypoventilation is bilateral degeneration of phrenic nerve motor neurons, which results in gradual weakening of the diaphragm [25,26]. During REM sleep, hypoventilation sets in and skeletal muscle activity, including the external intercostal and accessory respiratory muscles, is actively inhibited [27]. When diaphragmatic weakness occurs, there is insufficient alveolar ventilation to effectively exhale carbon dioxide (CO_2_). Considering that roughly 30% of individuals with evident hypercapnia will be overlooked if sleep tests solely include pulse oxymetry, capnographic instead of oxymetric methods are increasingly used to diagnose sleep-related hypoventilation in patients with neuromuscular disease or restrictive lung conditions [17,28]. Hypercapnia extends throughout non-REM sleep as diaphragm weakness worsens and eventually results in persistent hypercapnic respiratory failure. Sleep disturbances, such as stage transitions or intermittent arousals, can result from both hypercapnia and hypoxia [22]. 

Owing to the potential for decreased lung function, pulmonology work up of ALS patients should include tests of pulmonary function, including parameters such as forced vital capacity (FVC), a measurement of the maximum air volume expelled with the greatest amount of effort, and maximum inspiratory pressure (MIP), which represents the diaphragm’s and other inspiratory muscles’ strength [29]. These parameters are considered to be predictors of survival and ALS progression [30,31,32]. However, these parameters are seldom investigated in the prediction of sleep-disordered breathing [33,34]. In the early stages of disease, sleep-disordered breathing may not manifest, and sleep disturbance in ALS patients may be caused by sleep architecture fragmentation, which enhances the need for type one polysomnography (PSG) [35]. This test can accurately assess respiratory effort during sleep, identify different stages of sleep, detect anomalies in the electroencephalogram, and assess body position and limb movements [22]. This makes the assessment more helpful in identifying other sleep disturbances that also affect ALS patients, such as insomnia and periodic limb movement disorders, besides sleep-disordered breathing.

As such, this study aims to evaluate the overall sleep characteristics of patients with ALS (as well as the co-existence of sleep-disordered breathing), before the application of non-invasive ventilation (NIV) or continuous positive airway pressure (CPAP), their association with lung function tests, and possible predictor factors of survival.

## 2. Materials and Methods

### 2.1. Participants

A retrospective observational study was conducted among confirmed ALS patients redirected from a ventilation consultation from January 2009 to March 2024. ALS patients with hypoventilation symptoms (headache, orthopnea, non-restorative sleep, irritability, daytime somnolence) without muscular or respiratory functional impairment underwent PSG (no more than 3 months after respiratory function tests) by our instated pulmonology consultation protocol. Functional and sleep studies data were collected, as well as clinical and demographic information, by review of the clinical files. Patients under non-invasive ventilation before the PSG and patients who performed a cardiorespiratory polygraphy were excluded from the study. From a total of 169 ALS patients followed in the pulmonology consultation in this time frame, a total of 35 patients were included in the study.

This study was approved by the local ethics committee of a tertiary hospital center (n° 325/2016) (ULSTMAD).

### 2.2. Lung Function and Sleep Tests

All ALS patients performed respiratory functional tests, including a spirometry, with assessment while seated (st) and supine (sp), and maximal inspiratory (MIP) and expiratory pressure (MEP). Arterial gas exchange was also collected during the follow-up ventilation consultation every 3–6 months.

A diaphragmatic disfunction was assumed if FVCsp/FVCst > 12% [36].

Sleepware^®^ G3 4.01.0 software was used for the PSG analysis, and data were collected from left and right electro-oculograms, electroencephalography, chin and leg electromyography, pulse oximetry and heart rate, oronasal pressure and flow, chest and abdomen movement, body position, snoring, and EKG. Measurement of transcutaneous CO_2_ (TCM4 series, Radiometer^®^) was available in 10 patients. Sleep study data were analyzed and rescored according to the AASM criteria 3.0 [22]. In this study, we evaluated total apnea–hypopnea index (AHI), respiratory disturbance index (RDI), the percentage of recording time with SpO_2_ below 90% (T90), and the desaturation index. Nocturnal hypoventilation was defined if CO_2_ was ≥55 mmHg ≥ 10 min or if there was an increase of 10 mmHg or more from the awake baseline value to a value > 50 mmHg for at least 10 min [22]. 

Regarding the sleep parameters, we analyzed the sleep efficiency (%), number of sleep cycles, sleep latency (min), the percentage of REM sleep and N3 sleep, REM sleep latency (min), and the arousal index.

### 2.3. Statistical Analysis

Statistical analysis was performed using IBM SPSS^®^ 23. Descriptive data were reported as frequencies (*n*; %) and as the mean ± standard deviation or median (interquartile range—IQR) in the case of non-normally distributed data. Comparison between a continuous and categorical variable was performed by using the independent t-test and ANOVA or the Mann–Whitney U-test and Kruskal–Wallis test according to the normality distribution of the continuous variables. Correlations between continuous variables were analyzed using Pearson’s or Spearman’s correlation coefficient. Survival analysis was conducted by using Kaplan–Meier plots and the log-rank test. Cox regression analysis was used to predict the effect of different variables on survival. *p* values < 0.05 were considered statistically significant.

## 3. Results

### 3.1. Cohort Characteristics

A total of 35 ALS patients, in different stages of disease, underwent PSG and lung function testing, and their demographic and clinical characteristics are summarized in Table 1. 

The median time between the ALS diagnosis and the PSG was 9 months (IQR of 12).

A predominance of male gender was observed in the spinal onset patients, with no other demographic parameter showing a significant difference.

Regarding the lung function testing at the time of the PSG, our cohort showed a partially conserved FVC (74.88 ± 29.47%) with an impairment in MIP (46.72 ± 32.05) and MEP (57.86 ± 42.31). We observed a significantly lower MIP (*p* = 0.040) and MEP (*p* = 0.014) in bulbar onset patients, while FVC showed a similar tendency, although without statistical significance (*p* = 0.196).

When considering the sleep assessment, we observed a low median Epworth scale, 4.0 (4.0) pts, with the spinal onset patients showing a higher score (*p* < 0.001). 

Regarding the PSG, an overall diminished sleep efficiency (<80%) and REM sleep duration (<20%) was observed, along with an increased sleep (>30 min) and REM sleep latency (>120 min). Bulbar onset patients showed a trend for higher REM sleep latency, although without statistical significance. An overall median RDI of 10.20 (IQR 14.1) was observed, with 74.3% of these patients presenting with RDI ≥ 5/h. The overall desaturation index was 5.20 (12.20) and arousal index 16.30 (14.35). Eight patients (22.9%) met the hypoventilation criteria, with no difference among ALS onset type. Insomnia was observed in 15 patients (57.7%).

### 3.2. Lung Function Tests and PSG

**Figure 1:**
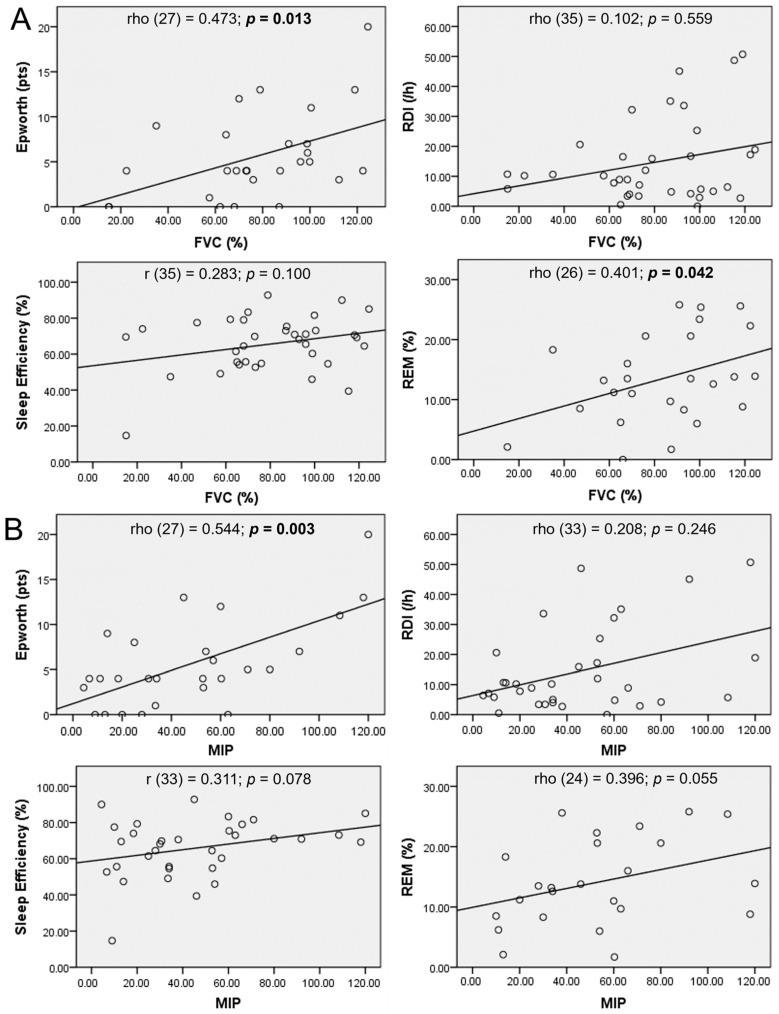
Correlation between lung functional and sleep parameters: Epworth sleepiness scale, respiratory disturbance index (RDI), sleep efficiency, and rapid eye movement (REM) sleep percentage. (**A**) Correlation between forced vital capacity (FVC) and sleep parameters. (**B**) Correlation between maximal inspiratory pressure (MIP) and sleep parameters.

A correlation analysis was conducted to examine the relationship between lung function and PSG parameters (Figure 1). An overall trend for positive correlation was observed between all features. A higher Epworth scale was significantly associated with a higher FVC (*p* = 0.013) and MIP (*p* = 0.003). A trend for higher RDI was also observed with a higher FVC (*p* = 0.559) and MIP (*p* = 0.246). The percentage of REM sleep was positively correlated with FVC and MIP, with statistical significance in FVC (*p* = 0.042).

When stratifying the lung function parameters (Table 2), an FVC below 50% showed a tendency for lower sleep efficiency and lower REM sleep percentage. The FVC 50–79% category showed a trend for better sleep quality, with a higher sleep efficiency and number of sleep cycles (*p* = 0.057) and lower REM sleep latency, RDI, arousal and desaturation index, T90, and hypoventilation criteria. The patients with an FVC ≥ 80% showed a tendency for a higher RDI as well as hypoventilation criteria.

A preserved MIP showed a trend for higher sleep efficiency and REM sleep (*p* = 0.088) and N3 percentage (*p* = 0.063). Paradoxically, it also showed a tendency for higher arousal, desaturation index, and hypoventilation criteria, with a significantly higher T90; however, when stratifying the MIP, most of the patients had a diminished value (28 vs. 5).

### 3.3. Survival Analysis

We observed an overall median survival of 36.00 months (CI 24.91 to 47.09).

**Figure 2:**
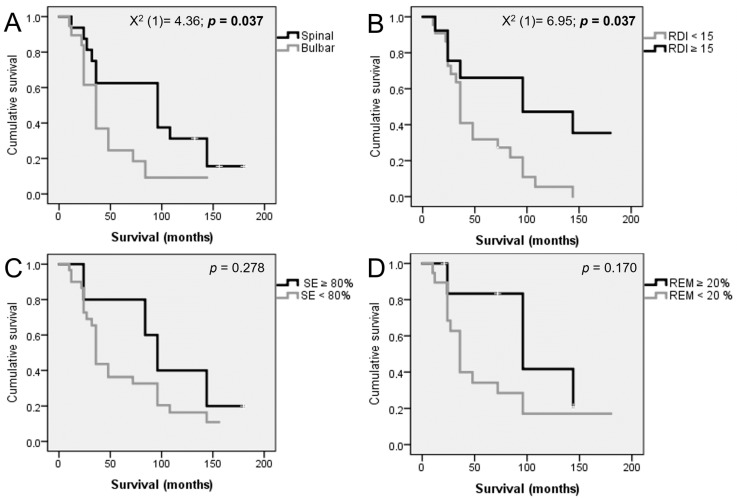
Kaplan–Meier survival analysis, according to ALS type of onset (**A**), RDI (**B**), SE—sleep efficiency, (**C**) REM sleep percentage, and (**D**) stratification.

A log-rank test was run to determine if there were differences in the survival distribution, according to the ALS onset type and RDI, sleep efficiency, and REM sleep percentage, after stratification (Figure 2). A spinal onset and RDI ≥ 15/h were associated with a significantly higher survival. A normal sleep efficiency (≥80%) and REM sleep (≥20%) also showed a trend for a higher survival, although without statistical significance.

A Cox regression was conducted to further assess the individual contribution of each sleep parameter in terms of survival. These variables statistically predicted survival: X^2^(3) = 8.913; *p* = 0.030. A normal REM sleep percentage and RDI ≥ 15/h were independent predictors of survival, but sleep efficiency did not add a statistical significance to the prediction.

## 4. Discussion

The presented cohort of ALS patients shows considerable sleep impairment, represented by the overall diminished sleep efficiency. From the analysis of Table 1, it becomes evident that this sleep disruption may not be fully attributed to the respiratory events but is strongly associated with ALS.

Considering the lung function tests, our cohort exhibited a partially conserved FVC, with a diminished MIP and MEP. When analyzing the relationship between lung function and sleep quality (including sleep efficiency above 80%, REM sleep percentage above 20%, and arousal index below 10/h), we observed a trend for higher sleep quality in patients with conserved function, with FVC showing significant correlation with REM sleep percentage. Few studies address this potential relationship [37], mostly focusing on subjective sleep parameters [38]. Those who address this show non-significant results due to low recruitment or the nature of retrospective studies.

In this study, a better sleep quality and higher RDI were associated with better overall survival. A possible cause for a better outcome in patients with RDI ≥ 15 could be the low percentage of bulbar onset patients in our cohort and the fact that an adequate treatment of the obstructive events is associated with better survival in other diseases, such as COPD [39]. This finding can also be found in Engel et al.’s and Georges et al.’s studies [40,41]. Other studies, however, have showed contradictory results. Quaranta et al. compared two groups of ALS patients, one with OSA and the other without OSA, and found a higher survival rate in those without OSA [42]; however, the patients included in this study showed no symptoms of hypoventilation and presented with a normal CO_2_ value. In contrast, in our cohort, the PSG was conducted once the patient presented with symptoms. Therefore, our study included patients of different stages of the disease when tested rather than at disease onset. This may explain the disparity between both studies.

Our study comes with considerable limitations, including a high selection bias, due to its retrospective nature. On the one hand, we only included patients referred to the ventilation consultation after the ALS diagnosis. On the other hand, as mentioned before, the PSG was conducted once the suspicion of sleep-related breathing disorder was brought up by the patients’ symptoms. As such, the absence of asymptomatic patients could also influence our results. Furthermore, due to the small number of patients in our investigation and the lack of capnography studies in many of them, the detection of hypoventilation may have been understated.

In our study, we did not adequately evaluate symptoms and quality of life scores, the clinical outcomes that most matter to the individual patient, namely, ALS patients.

In conclusion, our study indicates that ALS patients with sleep-related symptoms show significant sleep disturbance (sleep-disordered breathing or with other disturbances), highlighting the need for PSG in symptomatic patients with conserved lung function. It also revealed that lung function tests may be associated with sleep alterations. However, our cohort failed to find a cut-off in FVC and MIP which could predict the need for PSG in order to detection hypoventilation early. Nonetheless, our study allowed us to infer that a better sleep quality is associated with higher survival in ALS patients. A precocious diagnosis and adequate treatment of obstructive events also showed an association with better survival. Future research should therefore look at the reason behind the inefficiency in sleep, as well as potential therapeutic approaches for ALS patients. The best time to perform a PSG and how to determine when it is appropriate are still unanswered questions in the ALS diagnosis process.

## Figures and Tables

**Table 1:** Demographic, clinical, lung function, and type one polysomnography (PSG) characteristics, stratified by ALS type of onset. Average ± standard deviation; median (interquartile range). -Apnea hypopnea index (AHI), body mass index (BMI), forced vital capacity (FVC), maximal expiratory pressure (MEP), maximal inspiratory pressure (MIP), respiratory disturbance index (RDI), rapid eye movement (REM), percentage of recording time with SpO_2_ below 90% (T90).

	All Patients (n = 35)	Bulbar Onset (n = 19)	Spinal Onset (n = 16)	*p*
Male sex (n; %)	24; 68.60%	9; 47.40%	15; 93.80%	**0.003**
Age (years)	67.86 ± 13.26	68.42 ± 11.65	70.75 ± 13.09	0.581
Age at diagnosis (years)	62.05 ± 13.12	64.95 ± 11.75	63.25 ± 13.59	0.694
BMI (kg/m^2^)	25.94 ± 4.39	26.85 ± 5.11	26.59 ± 4.26	0.878
FVC (%)	74.88 ± 29.47	73.83 ± 30.75	86.80 ± 26.67	0.196
MIP (mmHg)	46.72 ± 32.05	35.36 ± 25.44	57.98 ± 34.96	**0.040**
MEP (mmHg)	57.86 ± 42.31	39.31 ± 28.05	75.94 ± 46.73	**0.014**
Timing after diagnosis (months)	9.0 (12.0)	6.0 (7.0)	14.0 (16.0)	**0.001**
Epworth (pts)	4.0 (4.0)	3.0 (4.0)	8.5 (8.0)	**<0.001**
Sleep efficiency (%)	66.73 ± 12.53	66.44 ± 10.12	67.10 ± 15.60	0.767
N° cycles	2 (2)	2 (1)	2 (2)	0.681
Sleep latency (min)	39.47 ± 21.41	40.71 ± 41.63	37.89 ± 18.31	0.896
REM latency (min)	168.50 (121.50)	187.50 (100.25)	125.50 (149.50)	0.403
N3 (%)	17.62 ± 18.45	16.70 ± 8.30	18.69 ± 9.94	0.583
REM sleep (%)	13.50 (11.95)	13.35 (11.68)	13.80 (12.10)	0.667
AHI (/h)	6.80 (13.70)	8.50 (14.62)	11.60 (29.50)	0.461
RDI (/h)	10.20 (14.1)	10.45 (16.70)	18.90 (27.40)	0.333
RDI ≥ 15/h (n; %)	13; 37.10%	5; 26.30%	8; 50.00%	0.149
Arousal Index (/h)	16.30 (14.35)	15.70 (15.13)	16.30 (16.40)	0.422
Desaturation Index (/h)	5.20 (12.20)	4.50 (6.85)	10.00 (19.40)	0.168
T90 (%)	0.50 (9.25)	0.25 (3.03)	1.00 (29.06)	0.344
Hypoventilation (n; %)	8; 22.9%	4; 21.10%	4; 25.0%	0.782

**Table 2:** Sleep parameters stratified by FVC and MIP categories. Average ± standard deviation; median (interquartile range). Forced vital capacity (FVC), maximal inspiratory pressure (MIP), respiratory disturbance index (RDI), rapid eye movement (REM), percentage of recording time with SpO_2_ below 90% (T90).

	**FVC < 50 (n = 7)**	**FVC 50-79 (n = 11)**	**FVC ≥ 80 (n = 17)**	***p***
Sleep efficiency (%)	60.88 ± 14.96	69.42 ± 12.73	67.21 ± 12.25	0.145
N° cycles	2 (2)	2.5 (3)	2 (2)	0.057
Sleep latency (min)	39.88 ± 19.71	44.48 ± 21.31	37.35 ± 22.90	0.335
REM latency (min)	152.25 (81.13)	146.75 (72.38)	201.50 (149.50)	0.767
N3 (%)	25.32 ± 7.35	17.47 ± 4.04	15.11 ± 9.71	0.083
REM sleep (%)	10.85 (13.33)	12.35 (7.35)	13.80 (14.60)	0.239
RDI (/h)	10.65 (7.83)	8.35 (14.38)	17.20 (30.30)	0.340
Arousal Index (/h)	16.30 (16.80)	13.10 (11.65)	16.80 (15.30)	0.837
Desaturation Index (/h)	6.00 (8.73)	3.40 (12.83)	5.80 (16.30)	0.815
T90 (%)	1.20 (16.45)	0.20 (7.57)	0.50 (14.90)	0.736
Hypoventilation (n; %)	2; 28.6%	1; 9.1%	5; 29.4%	0.422
	**MIP < 60 (n = 28)**	**MIP ≥ 60 (n = 5)**		***p***
Sleep efficiency (%)	64.91 ± 13.50	73.88 ± 6.43		0.229
N° cycles	2 (1)	3 (2)		0.197
Sleep latency (min)	37.57 ± 20.32	43.96 ± 28.35		0.699
REM latency (min)	170.50 (119.00)	105.50 (154.50)		0.271
N3 (%)	15.83 ± 8.40	23.46 ± 3.61		0.063
REM sleep (%)	12.60 (10.00)	20.60 (14.25)		0.088
RDI (/h)	10.60 (20.50)	18.90 (42.95)		0.248
Arousal Index (/h)	15.10 (18.90)	16.80 (9.45)		0.547
Desaturation Index (/h)	4.00 (8.50)	20.10 (48.50)		0.421
T90 (%)	0.30 (1.40)	45.20 (61.75)		**0** **.038**
Hypoventilation (n; %)	5; 17.9%	2; 40.0%		0.265

## Data Availability

The raw data supporting the conclusions of this article will be made available by the authors, without undue reservation, to any qualified researcher.
